# Fluoroquinolones’ Biological Activities against Laboratory Microbes and Cancer Cell Lines

**DOI:** 10.3390/molecules27051658

**Published:** 2022-03-03

**Authors:** Ghadeer A. R. Y. Suaifan, Aya A. M. Mohammed, Bayan A. Alkhawaja

**Affiliations:** 1 Department of Pharmaceutical Sciences, Faculty of Pharmacy, The University of Jordan, Amman 11942, Jordan; aya_a_mohammed@yahoo.com; 2 Department of Pharmacy, Faculty of Pharmacy and Medical Sciences, The University of Petra, Amman 11196, Jordan; bayan.alkhawaja@uop.edu.jo

**Keywords:** ciprofloxacin, moxifloxacin, norfloxacin, fluoroquinolones, resistant bacteria, anticancer, minimum inhibitory concentration

## Abstract

Development of novel derivatives to rein in and fight bacteria have never been more demanding, as microbial resistance strains are alarmingly increasing. A multitude of new fluoroquinolones derivatives with an improved spectrum of activity and/or enhanced pharmacokinetics parameters have been widely explored. Reporting novel antimicrobial agents entails comparing their potential activity to their parent drugs; hence, parent fluoroquinolones have been used in research as positive controls. Given that these fluoroquinolones possess variable activities according to their generation, it is necessary to include parent compounds and market available antibiotics of the same class when investigating antimicrobial activity. Herein, we provide a detailed guide on the in vitro biological activity of fluoroquinolones based on experimental results published in the last years. This work permits researchers to compare and analyze potential fluoroquinolones as positive control agents and to evaluate changes occurring in their activities. More importantly, the selection of fluoroquinolones as positive controls by medicinal chemists when investigating novel FQs analogs must be correlated to the laboratory pathogen inquest for reliable results.

## 1. Introduction

Antimicrobial prescriptions for the treatment of infections caused in particular by *Staphylococcus aureus (S. aureus)*, *Pseudomonas aeruginosa* (*P. aeruginosa*), and *Mycobacterium tuberculosis* (*M. tuberculosis*) have been affected by bacterial resistance [1]. Alarmingly, the ever-increasing emergence of resistant strains has globally increased the mortality rates [2]. 

**Figure 1:**
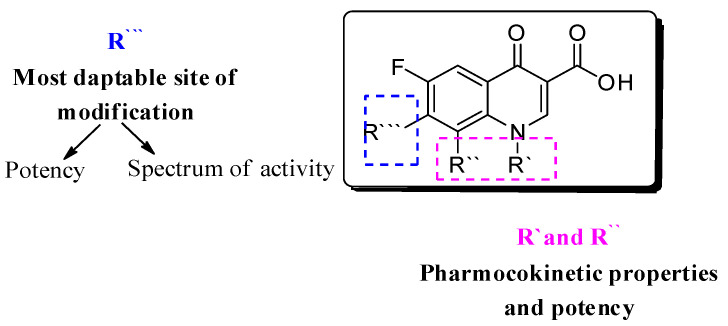
Fluoroquinolone’s nucleus: 1-substituted 1,4-dihydro-4-oxo-pyridine-3-carboxylic acid; R’, R’’ are responsible for pharmacokinetic properties, and R’’’ is responsible for potency.

Several approaches have been followed to develop novel fluoroquinolones (FQs) with enhanced antimicrobial activity and/or to enhanced pharmacokinetic properties to tackle bacterial resistance [3,4,5,6,7,8]. With more than 500 newly introduced structural modifications on FQs’ key scaffold [9]; 1-substituted 1,4-dihydro-4-oxo-pyridine-3-carboxylic acid (Figure 1) and the recent approval of delafloxacin in 2017, researchers have focused on embracing the biological activity of FQs, particularly against resistant bacterial strains [10,11].

Additionally, literature reviews pointed out FQs’ potential activities as anticancer, antitumor, antiviral, and antifungal agents in addition to their antibacterial activity where the latter is attributed to their ability to selectively inhibit bacterial type II topoisomerases, DNA gyrase, and/or topoisomerase IV [12,13,14,15].

Currently, FQs are one of the most widely used antimicrobial drugs, with a wide range of indications, covering respiratory infections, urinary tract infections (UTIs), gastrointestinal infections, and gynecologic infections [16]. Moreover, FQs are indicated as a prophylactic treatment in immune-compromised neutropenic patients [17]. 

**Figure 2:**
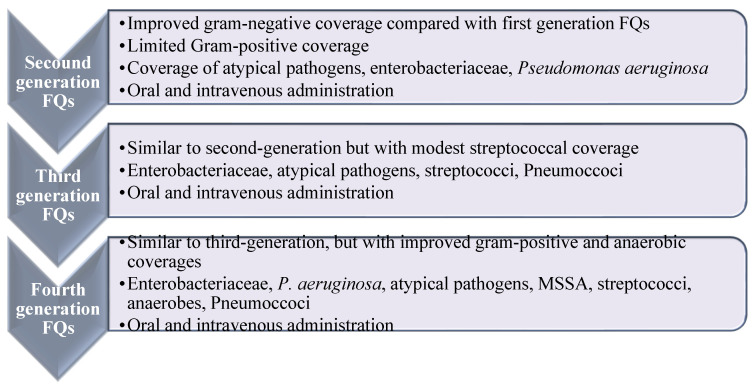
Spectrum and antimicrobial activities of fluoroquinolone based on their generations. Widening of the antibacterial activity of fluoroquinolones in relation to their generation. Reproduced/adapted from ref. [13].

FQs are usually classified into four generations with enhanced efficacy and spectrum of activity, along with enhanced safety and pharmacokinetic characteristics (Figure 2) [18,19]. Ciprofloxacin is the most prosperous derivative, both economically and clinically [20], and the newer generations such as levofloxacin, gemifloxacin, and moxifloxacin offer enhanced activity against aerobic Gram-negative bacilli and Gram-positive bacteria over ciprofloxacin, e.g., against *Streptococcus pneumoniae* (*S. pneumoniae*) and *S. aureus* [20]. Ciprofloxacin and moxifloxacin retain enhanced in vitro activity against *P. aeruginosa* [21]. In terms of potency, moxifloxacin is more potent against Gram-positive and anaerobes than ciprofloxacin and levofloxacin. Newer generations displayed potent activity against penicillin-resistant and multidrug-resistant (MDR) pneumococcus and anaerobic bacteria. Recently, delafloxacin was granted approval in 2017 for the systemic treatment of acute bacterial skin infections [22].

Appraisal of the newer FQs’ derivatives should be, in part, based on the relevant references. Herein, commonly employed FQ acting as positive controls in antimicrobial bioassays of up-to-date papers were reviewed. These results were reported in a constructive and comparative manner to facilitate the process of developing novel FQs’ analogues. The chemical structures and key physical properties of the frequently adopted standard FQs, namely norfloxacin **1**, ciprofloxacin **2**, levofloxacin **3,** and moxifloxacin **4** are summarized in Table 1. This should provide a facile referral guide to recent research areas concerning FQs derivatives antibacterial inhibitory effect, the adopted testing protocols, and generations-based comparison between different FQs to be applied in innovative research. Choosing standard FQs will not only affect the assessment of the new counterparts, but also provide a more comprehensive and efficient performance in assays.

## 2. Comparison of the In Vitro Antimicrobial Assays

A variety of methods and tactics could be adopted to evaluate the antibacterial activity of potential agents, and to draw constructive conclusions. In this regard, choosing and performing these assays varies according to the antimicrobial agents, availability of equipment, and cost-related reasons. The most known and basic standard methods are disk-diffusion [29] and broth or agar dilution methods [30]. The advantages and disadvantages of these assays are summarized in Table 2 and reviewed elsewhere [31,32], being apart from the scope of this article. In brief, standardized antimicrobial bioassays (antimicrobial susceptibility testing) are nowadays published and approved by the Clinical and Laboratory Standards Institute (CLSI) for bacteria and yeasts testing [33], herein the most commonly reported bioassays and the antimicrobial values of various FQs analogues are reported. 

Dilution methods afford quantitative evaluation of the in vitro antimicrobial activity, which are usually expressed as minimum inhibitory concentration (MIC) values and represent the lowest concentration of the tested antimicrobial agent that inhibits the visible growth of tested microorganism. A number of approved guidelines for dilution antimicrobial susceptibility testing of fastidious or non-fastidious bacteria, yeast, and filamentous fungi are reported [30]. 

On the other hand, agar disk-diffusion method is the standard qualitative method for routine antimicrobial susceptibility testing. This method provides qualitative results by categorizing bacteria as susceptible, intermediate, or resistant based on the obtained growth zones of inhibition (ZOI) diameters. However, important parameters, including the growth media, temperature, period of incubation, and the required inoculum size should be optimized to fulfil CLSI standards [22]. 

Differently, measuring the inhibition of supercoiling activity (catalytic activity) of DNA gyrase or the concentration of compounds required for inhibiting 50% of gyrase supercoiling activity (IC_50_) has been widely reported as an alternative assay to test the antibacterial activity of different FQs derivatives, particularly if the mechanistic and catalytical activity of the developed analogues are of concern [34,35]. 

## 3. FQ’s Antibacterial Biological Activity

### 3.1. FQ’s Antibacterial Activity against Gram-Positive Bacteria

According to the reviewed literature in the past five years, and for the sake of including up-to-date activities on the most common FQs applied as golden antimicrobial positive controls in laboratories, herein, standard FQs and their antimicrobial activity against a panel of laboratory microbes are reported (Table 3). 

As reported, norfloxacin was used as a positive control in the pipeline publications, including norfloxacin derivatives synthesis. Norfloxacin MIC against Gram-positive is presented in Table 3 [1,23,24,26,28,34,35,36,37,38,39,40,41,42,43,44,45,46,47,48,49,50,51,52,53,54,55,56,57,58,59,60,61,62,63,64,65,66,67,68,69,70,71]. In brief, norfloxacin inhibitory activity against a panel of Gram-positive bacteria regardless of the strain varied relatively. For example, norfloxacin in vitro antibacterial activity reported by Mentese et al. against *E. faecalis* ATCC 29212 varied from that reported by Seliem et al. (MIC ranged from <0.128 µM [46]−100.207 µM [47]). Similarly, norfloxacin MIC against *S. aureus* ATCC 25923 ranged from <0.128 µM [46]–156.170 µM [45] in the above-mentioned two different studies.

As illustrated in Table 3, ciprofloxacin was the most commonly adopted reference by the cited researchers against different Gram positive and negative bacterial stains, ciprofloxacin MIC against Gram-positive bacteria including *B. cereus* spp. ranged from 0.181 µM [46]−3.954 µM [28], *S. aureus* ATCC 6538 (ranged from 1.509 µM [48]−146.978 [49] µM), *S. aureus* ATCC 29213 (MIC ranged from 0.082 µM [67]−1.509 µM [48]), and *S. aureus* ATCC 25923 (MIC ranged from 0.010 [52] µM −3.954 µM [28]). Remarkably, ciprofloxacin MIC varied within similar bacterial species, one example is *S. epidermidis* species, according to Liu et al., strain MSSE 12-1 of *S. epidermidis* species was susceptible to ciprofloxacin (MIC 0.755 µM) [26], whereas it showed very limited activity against MSSE14-2 strain (MIC > 386.308 µM) [53,54]. Interestingly, discrepancy in MIC values was observed between similar bacterial strains as reported by different research groups with 100-fold MIC difference [48,49]. Minor variation between the adopted testing protocol for MIC determination, such as incubation temperature might be the driving factor for such a difference [48,49].

Considering the third FQ reference, levofloxacin was adopted by many researchers’ as a reference control, and exhibited variable antimicrobial activity against *E. faecalis* (MIC ranged from 1.384 µM for *E. faecalis* 51575 [55], 177.220 µM for *E. faecalis* ATCC 700221 [51]) as an example. A notable difference in levofloxacin potency against different *staph* strains, including methicillin-sensitive *S. aureus* (MSSA) [26,53,54,68,69], methicillin-resistant *S. aureus* (MRSA) [26,53,54,68,69], *S. epidermidis*, and *S. pneumoniae* was observed (Table 3).

Following scientific reports in the literature, levofloxacin exhibited superior antibacterial activity against Gram-positive *S. epidermis* strains [51,55,63] and moxifloxacin is generally the most potent amongst FQs a, gainst Gram-positive and negative bacteria [26]. Moxifloxacin was the latent agent against the food poisoning pathogen *L. monocytogenes* ATCC 43251 (MIC < 1.370 µM [28]) when compared with other FQs as ciprofloxacin (MIC 3.954 µM−12.072 [28,64]) and norfloxacin (MIC < 8.267 µM [28]).

### 3.2. FQs Antibacterial Activity against Gram-Negative Bacteria

A summary of common laboratory tested Gram-negative bacteria and standard fluoroquinolones antibiotics are presented in Table 4. It is noticeable that ciprofloxacin has potential antibacterial activity against Gram-negative bacteria as *P. aeruginosa* and *E. coli*. [28,48]. Moreover, ciprofloxacin had prospective growth inhibitory activity against *H. pylori* NCTC 11916 and 12 more *H. pylori* clinical isolates as reported by Abu-Sini et al. [72]. Ciprofloxacin broad spectrum of activity against aerobic and anaerobic Gram-negative bacteria is shown in Table 4. 

Nevertheless, Gorityala et al. [56] reported that ciprofloxacin potency against *P. aeruginosa* were superior compared to moxifloxacin. This pattern was also noticed in results published by Türe et al. and Garza et al., [28,50].

Norfloxacin inhibitory activity against a panel of Gram-negative bacterial type, and on the same bacterial strain is noted to be varied. For instance, norfloxacin in vitro antibacterial activity reported by Pardeshi et al. against *E. coli* ATCC 25922 varied from that reported by Leyva-Ramos et al. (MIC ranged from < 0.094 µM [24]−117.433 µM [45]). Moreover, norfloxacin and ciprofloxacin MIC against different *P. aeruginosa* strains ranged from 1.002 µM [1]−1565.773 µM [45] and <0.091 [62] µM−150.901 µM [42], respectively, in different studies. On the contrary, ciprofloxacin MIC against a panel of Gram–negative pathogens looks more consistent (*A. haemolyticus* ATCC 19002 (MIC 0.755 µM) [62], *A. baumannii* ATCC17961 (MIC 0.24 µM) [58], *A. calcoacetious* ATCC 19606 (MIC 1.509 µM) [55], and *C. freundii* ATCC 43864 (MIC 1.38 µM) [51]. However, a wide range in ciprofloxacin MIC against *E. coli* ATCC 25922 is perturbing as MIC reported ranged from 0.002 µM [24]−61.869 µM [49] in different publications. This fluctuation in ciprofloxacin antibacterial activities may explain the current abundant application of levofloxacin and moxifloxacin as positive standards by medicinal chemists when designing and synthesizing novel FQs analogues [24,28,53,54,55,68,69,70,73,74,75].

As presented in Table 4, different studies reported the use of third generation levofloxacin as a positive control against a wide range of Gram-negative organisms includes *P. aeruginosa*. For this infectious pathogen, MIC ranged from 5.453 µM [68] for *P. aeruginosa* 14–19 strain to 87.241 µM [69] for *P. aeruginosa* 12–14 strain. Similarly, levofloxacin MIC against *K. pneumonia* ranged from 0.082 µM [69] for *K. pneumonia* 12–4 strain to 87.241 µM [54] for *K. pneumonia* 14–3 strain. According to Zhang et al., [69] levofloxacin is around five hundred time more potent against *K. pneumonia* 12–4 strain compared to *P. aeruginosa* 12–14 strain, though both are Gram-negative pathogens. However, in another by Huang et al. [68], levofloxacin was more potent against *P. aeruginosa* for 14–19 strain compared to *K. pneumonia* for 14–2 strain. It is worth mentioning that the bacterial strain is the variant factor in both articles. This indeed highlights the importance of referring to the relevant standard control during laboratory investigation and comparisons.

A similar pattern of the wide range of MIC values against the same strain was observed, where the MIC of norfloxacin against *E. coli* ATCC-25922 ranged from <0.094 µM [24] to 117.433 µM [45]. 

### 3.3. FQs’ Antimycobacterial Activity

FQs, particularly ciprofloxacin was included as a positive control along with isoniazid and rifampicin against various Mycobacterium strains as shown in Table 5 [24,26,27,28,58,63,65,68,75,81,82]. Furthermore, levofloxacin in vitro anti-mycobacterial activity was reported and found to be comparable to ciprofloxacin [26,68]. Recent studies by Hu et al., [82] and Mohammed et al., [65] declared moxifloxacin in vitro anti-mycobacterial activity to be more potent than both ciprofloxacin **1** and levofloxacin **3**.

### 3.4. FQs’ Antifungal, Antiparasitic, and Anticancer Activity

Apart from their antibacterial activity, FQs were also tested for their antifungal activity with little effect on most fungi. Since the late 1980s, studies revealed anti-trypanosomal activity for the quinolones prototype, nalidixic, and oxolonic acid derivatives [14]. Other studies illustrated the antiparasitic activity of norfloxacin against Plasmodium falciparum and the inhibitory effect of other fluoroquinolones against Plasmodium family [14,83,84]. Today, quinolone-amides related derivatives were used to design anti-trypanosomal compounds with many of them presenting potential in vivo activity [85]. 

Anticancer activity of FQs were also evaluated against a range of cancer cell lines, such as A549 Lung adenocarcinoma, HCT-116 colon cancer, MCF-7 breast cancer cell lines, and others have been determined previously and compared with the developed counterparts [48,50,61,66] as presented in Table 6.

### 3.5. FQs Inhibitory Effect as Anti-Viral Agaents against SARS-CoV-2 and HIV-1

As researchers investigate several approaches to combat COVID-19 infection, there is a wide interest in fluoroquinolones. Ciprofloxacin and Moxifloxacin were tested through in silico molecular docking and showed the potential binding capacity to SARS-CoV-2 main protease (M^pro^) and low binding energy. Moreover, a recent study evaluated the potency and cellular toxicity of selected FQs (enoxacin, ciprofloxacin, levofloxacin, and moxifloxacin) against SARS-CoV-2 and MERS-COV. This study showed that a high concentration of the tested FQs should be employed to prevent viral replication with enoxacin being the superior (EC50 of 126.4) against SARS-CoV-2 [14,83,84]. Other studies evaluated FQs anti-HIV-1 activities. However, FQs standards activity were not presented [65].

## 4. Recommendations

Based on recently published research where FQs were used as positive controls against several microorganisms and cancer cells, it is recommended to use the most active FQ in future studies in addition to the parent drugs to compare the benefits and to have an accurate insight when reporting results.

The difference perceived in FQs’ potency according to different research articles is challenging and could be attributed to several factors, including the different testing protocols implemented by each research group, solvents or broth used in bacterial culturing, incubation time, bacterial concentration tested, bacterial growth phase, reader instrument sensitivity, etc.

Ciprofloxacin is recommended to be used as a control against Gram-negative bacteria whether resistant or susceptible. If mainly Gram-positive activity is concerned, levofloxacin or moxifloxacin might be the best choices. The wide-spectrum and potent newer generations should be compared with, when broader comparison is desired. Choose moxifloxacin if the development of newer FQs derivatives is not a biologically-based design. This should provide a proper perspective when reporting novel FQs and their activities. Working against Mycobacterium stains, moxifloxacin was found to be more active compared to the other FQs, thus it is advisable to consider it as a positive control.

Moreover, the authors spur adopting preliminary activity testing of the chosen strains before commencing biological evaluation of interest as some of the stains might not be susceptible to the reference drugs. Lastly, given that some stains exhibited varied MIC values against the same drug, we recommend revising the adopted protocols beforehand to get more accurate comparable results of the reference drug, which will be then more reliable to base the conclusions upon.

## Figures and Tables

**Table 1:** Most adopted standard fluoroquinolones, their chemical structures, and key physical properties.

Fluoroquinolone	Structure	Generation	Physical Properties	References
**Norfloxacin**	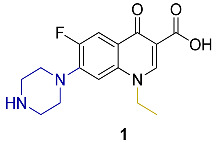	2nd	*ClogP* 1.81	[23,24]
**Ciprofloxacin**	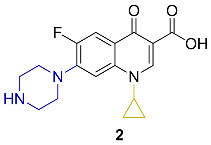		Log*P*_exp_–0.1432	[23,25,26,27]
			*ClogP*–0.725	
			*ClogP* 1.32	
			*ClogP* 1.55	
**Levofloxacin**	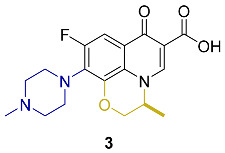	3rd	*ClogP* 1.35 *ClogP*–0.51	[24,26]
**Moxifloxacin**	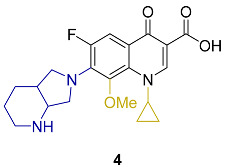	4th	*ClogP* 2.53 Log*P* 1.60	[24,28]

**Table 2:** Advantages and disadvantages of commonly applied technique for the evaluation of drugs antimicrobial activity.

Testing Technique	Advantages	Disadvantages	Reference
Disk-diffusion	- Can be used to for routine susceptibility testing - Ability to adjust the tested discs - Simple - Standardized - Low cost - Reproducible	- Diffusability of drug from disc must be considered - Results are qualitative - Requires large inoculum size 1–2 × 10^8^ CFU/ mL - Can only approximate MIC based on diameter of the zones of inhibition	[36,37]
Dilution methods	- Includes agar dilution, broth microdilution and broth macrodilution methods - Can be used to accurately calculate MIC against various bacteria, yeasts, and fungi - Can be used to monitor resistance emergence - Reproducible - Low cost - Can test multiple bacteria in one platex using agar dilution method - Agar dilution method can be semi-automated	- Broth macrodilution has higher risk of error- Broth microdilution may not detect contamination, inoculum viability and the inhibitory effect of cosolvents used (e.g., dimethyl sulphoxide)- Agar dilution method requires intense labor and high cost unless it is automated	[31,38]

**Table 3:** Fluoroquinolones’ antibacterial activity against Gram-positive bacterial strains.

Fluoroquinolone		G +ve Bacteria	Strain	MIC (µM)	Reference
Generation	Name				
Second Generation	Norfloxacin	*B. subtilis*	NCDC 71	15.658	[42]
		*B. cereus*	8035	4.697	[44]
			Roma 702	<0.128	[46]
			Roma 709	8.267	[28]
		*B. polymyxa*	NCDC 64	78.289	[42]
		*E. faecalis*	ATCC 29212	<0.128	[46]
				8.267	[28]
				100.207	[47]
		*L. acidophilus*	RSKK 06029	2113.794	[28]
		*L. monocytogenes*	ATCC 43251	8.267	[28]
		*S. aureus*	NCDC 110	31.315	[42]
			ATCC 29213	3.132	[43]
			ATCC 25923	156.170	[45]
				4.134	[28]
				<0.128	[46]
			*S. aureus* 209p	1.221	[44]
			MRSA	1.879	[28]
		*S. pneumonia*	ATCC 49619	19.572	[43]
	Lomefloxacin	*B. cereus*	8035	17.931	[44]
		*S. aureus*	209p	2.220	
	Ciprofloxacin	*A. baumannii*	24.144		[24]
			ATCC 19606	2.354	[50]
		*B. cereus*	ATCC 10876	0.360	[57]
			Roma 702	0.181	[46]
			Roma 709	3.954	[28]
		*B. polymyxa*	NCDC 64	30.180	[42]
		*B. subtilis*	ATCC 6633	0.090	[57]
				0.030	[58]
				8.149	[34]
			NCDC 71	60.361	[42]
				72.433	[59]
		*E. Faecalis*	ATCC 29212	3.018	[56,60,61]
				1.360	[51]
				0.368	[46]
				1.509	[55,62]
				7.878	[28]
			ATCC 33186	2.384	[50]
			ATCC 51575	1.360	[51]
			ATCC 51299	1.509	[55]
			JH2-2	6.036	[63]
			UCN41	3.018	[63]
			*E. faecalis*	24.144	[47]
			14-1	96.577	[53,54]
			14-2	3.018	[53,54]
		*E. faecium*	ATCC-19434T	3.018	[63]
			BM-4147	12.072	[63]
			ATCC 27270	2.651	[56]
			ATCC 700221	>386.308	[55]
			13-7	>386.308	[55]
			14-2	96.577	[53,54]
			14-5	386.308	[53,54]
			14-6	>386.308	[53,54]
		*E. hirae*	ATCC 10541	24.144	[48]
		*K. pneumonia*		193.154	[24]
		*L. acidophilus*	RSKK 06029	252.277	[28]
		*L. monocytogenes*	ATCC 43251	3.954	[28]
			EGD	12.072	[64]
			CLIP21369	48.288	[64]
		*S. aureus*	ATCC 6538	26.015	[65]
				0.800	[66]
				146.978	[49]
				1.509	[48]
			ATCC 29213	0.400	[66]
				1.509	[48]
				0.082	[67]
				1.509	[60,61]
				0.296	[50]
				0.680	[51]
				0.755	[55]
			ATCC 25923	0.755	[64]
				2.960	[57]
				0.010	[52]
				0.755	[26]
				0.368	[46]
				3.954	[28]
				3.018	[62]
			*S. aureus* ATCC 25923 (clinical isolate)	0.755	[63]
			SAI	24.144	[64]
			*SAI24*	48.289	[64]
			*SA036*	96.577	[64]
			N41120032	193.154	[64]
			SG511	0.470	[58]
			Microbank 14001 (MRSA)	1.480	[57]
			*S. aureus D15* MRSA	3.100	[66]
			*S. aureus D17* MRSA	3.100	[66]
			*S. aureus* CIP^R^	50.000	[66]
			*S. aureus* NCTC 4163	0.755	[48]
			*S. aureus* HG001 (laboratory strain)	0.377	[63]
			MSSA 12-1	0.755	[26]
			MSSA 12-2	0.755	[26]
			MSSA 12-4	0.755	[26]
			MSSA 12-5	0.755	[26]
			MSSA 14-1	96.577	[53,54]
			MSSA14-3	0.377	[53,54]
			MSSA 14-4	1.509	[53,54]
			MRSA	3.954	[28]
			MRSA 14-4	>386.308	[53,54]
			MRSA 14-5	48.288	[53,54]
			MRSA 12-2	193.154	[26]
			MRSA 12-4	193.154	[26]
			MRSA 12-5	96.577	[26]
			CMCC 26003	1.509	[53,54]
			*S. aureus* ATCC 700699 (resistant isolate)	>24.144	[63]
			Healthcare-acquired MRSA NRS70	0.604	[50]
			Community-acquired MRSAUSA300	19.014	[50]
			(MRSA) ATCC 33591	1.509	[60,61]
				0.755	[55]
				0.680	[51]
			MRSA ATCC 33592	≤0.083	[56]
			NCDC 110	150.901	[42]
			12.072		[47]
			0.589		[49]
			0.377		[24]
		*S. epidermidis*	ATCC 12228	0.400	[66]
				1.480	[57]
				0.755	[48]
			ATCC 14990	0.377	[63]
			ATCC 35984	≤0.181	[63]
			*-*	0.589	[49]
			MSSE CANWARD-2008 81388	≤0.083	[56]
			MSSE ATCC 12228	0.377	[55]
				0.340	[51]
			MSSE 12-1	0.755	[26]
			MSSE12-3	6.036	[26]
			MSSE12-6	0.755	[26]
			MSSE12-8	12.072	[26]
			MSSE14-2	>386.308	[53,54]
			MRSE CAN-ICU 61589 (CAZ > 32)	42.411	[56]
			MRSE12-1	24.144	[26]
			MRSE12-6	48.288	[26]
			MRSE 13-3	193.154	[55]
			MRSE14-21	193.154	[54]
			MRSE14-22	386.308	[53,54]
			MRSE14-37	386.308	[53,54]
			MRSE14-39	386.308	[53,54]
			MRSE 16-3	32.897	[54]
		*S. pneumoniae*	ATCC 19615	6.036	[54]
			ATCC 49619	0.331	[56]
			R6	1.177	[50]
	Cipro HCl	*B. cereus*	Roma 709	1.636	[28]
		*E. faecalis*	ATCC 29212	3.435	[28]
		*L. acidophilus*	RSKK 06029	219.385	[28]
		*L. monocytogenes*	ATCC 43251	3.435	[28]
		*S. aureus*	ATCC 25923	6.843	[28]
			MRSA	3.435	[28]
Third Generation	Levofloxacin	*E. faecalis*	ATCC 29212	2.770	[51]
				2.767	[55]
			ATCC 51575	1.380	[51]
				1.384	[55]
			ATCC 700221	177.220	[51]
			14-1	44.276	[68]
				354.210	[53,54]
			14-2	88.552	[68]
				2.767	[53,54]
			14-3	177.104	[68]
		*E. faecium*	ATCC 700221	88.552	[55]
			13-7	88.552	[55]
			14-1	354.210	[68]
			14-2	88.552	[53,54]
			14-2	2.767	[68]
			14-5	177.105	[53,54]
			14-6	177.105	[53,54]
			16-4	44.300	[51]
		*S. aureus*	ATCC 25923	<0.022	[26]
				0.166	[69]
			ATCC 29213	0.350	[55]
				0.350	[51]
			CMCC 26003	0.346	[68]
				0.346	[53,54]
			MSSA12-2	0.346	[26]
			MSSA 12-4	0.166	[69]
				0.344	[26]
			MSSA12-5	0.346	[26]
			MSSA14-1	22.138	[53,54]
			MSSA 14-2	0.692	[68]
			MSSA14-3	0.346	[53,54,68]
			MSSA14-4	1.384	[53,54,68]
			MRSA 12-1	177.105	[69]
			MRSA12-2	88.552	[26]
			MRSA12-4	88.552	[26]
			MRSA12-5	88.552	[26]
			MRSA14-4	177.105	[53,54,68]
			MRSA14-5	22.138	[26,53,54]
			NARSA 10198	88.552	[70]
			NARSA 10193	88.552	[70]
			ATCC 29213	1.384	[70]
		*S. epidermidis*	MSSE ATCC 12228	0.350	[51]
				0.346	[55]
			12-1	0.346	[26]
			12-3	1.384	[26]
			12-6	0.346	[26]
			12-8	11.069	[26]
			12-1	11.069	[26]
			12-6	88.552	[26]
			MRSE12-1	0.083	[69]
			MSSE14-2	>354.210	[53,54]
				354.210	[68]
			MSSE12-3	1.384	[69]
			MSSE14-4	2.767	[68]
			MSSE14-6	5.534	[68]
			MRSE 13-3	88.552	[55]
			MRSE14-21	177.105	[53,54]
			MRSE14-22	88.552	[53,54,68]
			MRSE14-37	177.105	[53,54,68]
			MRSE14-39	177.105	[53,54,68]
			MRSE 16-3	5.540	[51]
		*S. pneumoniae*	ATCC 49619	0.346	[69]
			ATCC 19615	1.384	[53,54,68]
	Sparifloxacin	*B. cereus*	8035	0.484	[44]
		*S. aureus*	209p	0.484	
	Gatifloxacin	*B. subtilis*	NCDC 71	213.109	[42]
		*B. polymyxa*	NCDC 64	26.639	[42]
		*S. aureus*	NCDC 110	13.319	[42]
			ATCC 29213	0.333	[71]
			MSSA clinical isolates	0.333	[71]
			MRSA clinical isolates	42.622	[71]
		*S. epidermidis*	ATCC 12228	0.160	[71]
			MSSE clinical isolates	0.160	[71]
			MRSE clinical isolates	0.160	[71]
	Moxifloxacin HCl	*B. cereus*	Roma 709	<1.370	[28]
		*E. faecalis*	ATCC 33186	0.891	[50]
			14-1	18.296	[68]
			14-2	36.539	[68]
			14-3	18.296	[68]
		*E. faecium*	ATCC 29212	<1.370	[28]
			14-1	73.077	[68]
			14-2	1.142	[68]
			MSSE 12-3	0.284	[26,69]
			MSSE 12-6	0.069	[26]
			MSSE 12-8	2.284	[26]
			MSSE 14-4	4.567	[68]
			MSSE 14-6	4.567	[68]
			MRSE 12-1	0.571	[26,69]
			MRSE 12-6	16.539	[26]
			MRSE 14-22	18.269	[68]
			MRSE 14-37	18.269	[68]
			MRSE 14-39	18.269	[68]
		*L. acidophilus*	RSKK 06029	92.785	[28]
		*L. monocytogenes*	ATCC 43251	<1.370	[28]
		*S. aureus*	ATCC 25923	2.900	[28]
				<0.018	[26,69]
			CMCC 26003	0.137	[68]
			MSSA ATCC 29213	0.057	[50]
			MSSA12-1	0.034	[26]
			MSSA12-2	0.018	[26]
			MSSA12-4	<0.018	[26,69]
			MSSA12-5	0.034	[26]
			MSSA14-3	<0.018	[68]
			MSSA14-4	<0.018	[68]
			community-acquired MRSAUSA300	3.654	[50]
			healthcare-acquired MRSA NRS70	0.057	[50]
			MRSA 12-1	18.269	[69]
			MRSA12-2	18.269	[26]
			MRSA12-4	18.269	[26]
			MRSA12-5	18.269	[26]
			MRSA 14-4	27.404	[68]
			MRSA14-5	18.269	[68]
			MRSA	<1.370	[28]
		*S. pneumoniae*	ATCC 19615	0.034	[68]
			ATCC 49619	0.137	[69]
			R6	0.365	[50]

*Acinetobacter baumannii* (*A. baumannii*); American Type Culture Collection (ATCC); *Bacillus cereus* (*B. cereus*); *Bacillus polymyxa* (*B. polymyxa*); *Bacillus subtilis* (*B. subtilis*); China Center of Industrial Culture Collection (CMCC); *Enterococcus faecalis* (*E. faecalis*); *Enterococcus faecium* (*E. faecium*); *Enterococcus hirae* (*E. hirae*); *Klebsiella pneumonia* (*K. pneumonia*); *Lactobacillus acidophilus* (*L. acidophilus*); *Listeria monocytogenes* (*L. monocytogenes*); Methicillin-resistant *staphylococcus aureus* (MRSA); Methicillin-resistant *staphylococcus epidermidis* (MRSE); Methicillin-sensitive *staphylococcus aureus* (MSSA); Methicillin- sensitive *staphylococcus epidermis* (MSSE); Nigeria Centre for Disease Control (NCDC); *Staphylococcus aureus* (*S. aureus*); *Staphylococcus enterica* (*S. enterica*); *Staphylococcus epidermidis* (*S. epidermidis*); *Streptococcus pneumoniae* (*S. pneumoniae*).

**Table 4:** Fluoroquinolones’ antibacterial activity against Gram-negative bacterial strains.

Fluoroquinolone		G −ve Bacteria	Strain	MIC (µM)	Reference
Generation	Name				
Second Generation	Norfloxacin	*E. coli*	ATCC 8739	<0.251	[1]
			ATCC 25922	3.132	[43]
				<0.094	[24]
				<1.879	[28]
				117.433	[45]
				0.128	[46]
			ATCC 35218	6.263	[46]
			F-50	0.595	[44]
			NCDC 134	125.262	[42]
		*K. pneumoniae*	ATCC13883	4.134	[28]
		*P. aeruginosa*	ATCC 9027	9.708	[44]
				1.002	[1]
			ATCC 27853	>1565.773	[45]
				19.572	[43]
			ATCC 43288	16.503	[28]
			NCDC 105	46.973	[42]
			PAO1	12.526	[47]
		*Y. pseudotuberculosis*	ATCC 911	1.879	[28]
				0.128	[46]
	Lomefloxacin	*E. coli*	F-50	8.823	[44]
		*P. aeruginosa*	9027	17.931	[44]
	Ciprofloxacin	*A. haemolyticus*	ATCC 19002	0.755	[62]
		*A. baumannii*	ATCC17961	0.240	[58]
			CIP 7010	0.377	[62]
			CAN-ICU 63169	6.036	[21]
		*A. coacetius*	ATCC 19606	1.509	[55]
				1.360	[51]
		*C. freundii*	ATCC 43864	≤0.091	[55]
				1.380	[51]
		*E. aerogenes*	ATCC 13048	≤0.080	[51]
				≤0.091	[55]
		*E. cloacae*	ATCC 43560	≤0.091	[55]
				≤0.080	[51]
		*E. coli*	ESBLs(+)14-11	24.144	[54]
				48.289	[55]
			ESBL^+^ 14-2	96.577	[54]
			14-1	24.144	[54]
			14-2	24.144	[54]
			ATCC-29213	≤0.755	[21,52]
			ATCC 25922	<1.811	[28,63]
				0.024	[54,57]
				0.031	[48]
				0.010	[66]
				61.869	[49]
				0.091	[62]
				0.002	[24]
			NR 17663	0.002	[24]
			NR 17666	0.045	[24]
			NR 17661	96.577	[24]
			ATCC 25922 ESBLs(-);	≤0.091	[55]
				≤0.080	[51]
			ATCC 25922 (wild type)	≤ 0.091	[76]
			ATCC 35218	0.045	[60,61]
				16.961	[34]
				≤0.080	[51]
			BW5328/pAH69 (wild type)	≤ 0.091	[76]
			CAN-ICU 61714 (GEN-R)	≤0.755	[21]
			CAN-ICU 63074 (AMK 32)	≤0.755	[21]
			CANWARD-2011 97615	772.616	[21]
			gyrA S83LD87N, parC S80I E84G, AcrA+	>96.577	[76]
			DC0	0.470	[58]
			DC2	0.240	[58]
			F-50	0.573	[44]
			K12	0.604	[50]
			K12 Δ*lacU169*	0.005	[67]
			K12 Δ*lacU169 tolC::*Tn*10*	0.001	
			K12 Δ*lacU169 tolC::*Tn*10 gyrA* S83L	0.019	
			K12 Δ*lacU169 tolC::*Tn*10 gyrA* D87Y	0.009	
			imp-4213 (permeable outer membrane)	≤0.091	[76]
			JW5503-1 (ΔtoIC)	≤0.0.091	[76]
			MC4100 (wild type)	≤0.091	[76]
			NB27005-CDY0039 (ΔtolC, gyrA S83L D83G, parC S80I)	6.036	[76]
			NCDC 134	75.451	[42]
			NCTC 8196	0.031	[48]
				0.040	[66]
			ATCC 8739	28.007	[65]
			Penicillin Resistant *E. coli*	0.377 μM (68.9% survival of bacteria	[77]
		*H. pylori*	NCTC 11916	1.811	[72]
			Clinical isolate	0.905	[72]
		*K. pneumoniae*	ATCC 13883	≤0.755	[21]
				1.811	[28]
				0.755	[62]
				0.050	[66]
			ATCC 35657	0.021	[60,61]
			ATCC 700603 ESBLs (+)	1.509	[55]
				1.360	[51]
				0.755	[63]
			7 ESBLs(-)	≤0.091	[55]
			7 ESBLs (-)	≤0.080	[51]
			ESBL^+^ 14–17	1.509	[54]
			ESBL^+^ 14–18	1.509	[54]
			ESBL^+^ 14–19	193.154	[54]
			14-1	96.577	[54]
			14-2	48.288	[54]
			14-3	>386.308	[54]
			14-4	96.577	[54]
			*K. pneumonia*	40.160	[78]
		*M. catarrhalis*	ATCC 25238	0.091	[60,61]
		*M. morganii*	ATCC 25830	≤0.091	[55]
				≤0.080	[51]
		*P. aeruginosa*	ATCC 9027	0.720	[57]
				1.177	[44]
			ATCC 15442	0.755	[48]
			ATCC 43288	<0.091	[62]
				3.954	[28]
			ATCC 27853	1.509	[48]
				1.509	[54]
				0.680	[51]
				0.755	[55]
				0.755	[62,63]
				3.018	[21]
			CAN-ICU 62308 (GEN-R)	6.036	[21]
			CANWARD-2011 96846	12.072	[21]
			DSM 1117 Mueller–Hinton	0.755	[79]
			DSM 1117 Succinate minimum medium	0.755	
			DSM 1117 Succinate minimum medium + FeCl_3_ (1 lM)	0.755	
			AM 85 Mueller–Hinton	48.288	
			AM 85 Succinate minimum medium	48.288	
			AM 85 Succinate minimum medium + FeCl3 (1 lM)	96.577	
			K799/wt	0.470	[58]
			K799/61	0.240	[58]
			K1542 (ΔmexX, DmexB)	0.181	[76]
			NCDC 105	150.901	[42]
			NB52023-CDK005 (ΔmexX, DmexB, gyrA T83I)	1.509	[76]
			NB52023-CDK006 (ΔmexX, ΔmexB, gyrA T83I, parC S87L)	12.072	[76]
			PAO1	1.177	[50]
			PA01 (Wild type)	0.377	[76]
			*-*	5.030	[47]
			-	0.589	[49]
			14-9	1.509	[54]
			14-14	3.018	[54]
			14-15	3.018	[54]
			14-16	3.018	[54]
		*P. mirabilis*	ATCC 12453	0.045	[57]
			ATCC 49565	≤0.080	[51]
			13-1	≤0.091	[55]
		*P. rettgeri*	ATCC 31052	≤0.091	[55]
				≤0.080	[51]
		*P. vulgaris*	ATCC 29905	≤0.091	[55]
				≤0.080	[51]
		*S. marcescens*	ATCC 21074	0.160	[51]
				0.181	[55]
		*S. maltophilia*	ATCC 13636	5.450	[51]
				12.072	[55]
			CAN-ICU 62584	1.325	[56]
		*S. pneumoniae*	ATCC 49619	0.755	[26]
			12-18	3.018	[26]
		*Y. pseudotuberculosis*	ATCC 911	1.812	[28]
	Ciprofloxacin HCl	*E. aerogenes*	ATCC 13048	0.086–0.172	[64]
			CM64	01.363	[64]
		*E. coli*	ATCC 25922	<1.636	[28]
				0.022 (pH 7.4)	[64]
		K. pneumoniae	ATCC13883	<1.636	[28]
		*P. aeruginosa*	ATCC 43288	3.435	[28]
		*Y. pseudotuberculosis*	ATCC 911	<1.636	[28]
Third Generation	Levofloxacin	*A. coacetious*	ATCC 19606	0.346	[55]
				0.350	[51]
		*C. freundii*	ATCC 43864	≤.0.083	[55]
				≤0.080	[51]
		*E. aerogenes*	ATCC 13048	0.166	[55]
				0.170	[51]
		*E. cloacae*	ATCC 43560	≤.0.083	[55]
				≤0.080	[51]
		*E. coli*	ATCC 25922	0.346	[68]
				0.0412	[24]
				<0.022	[69]
			ATCC 25922 ESBLs^−^	≤0.083	[55]
				88.610	[51]
			ATCC 35218 ESBLs^+^	≤0.080	[51]
			NR 17663	0.083	[24]
			NR 17666	0.083	[24]
			NR 17661	88.552	[24]
			12-6	0.692	[69]
			12-11	11.069	[69]
			ESBL^+^ 14-1	11.069	[54]
				44.276	[69]
				5.534	[68]
			ESBL^+^ 14-2	21.810	[54]
				21.810	[68]
			14-1	21.810	[54]
				10.905	[68]
			14-2	21.810	[54]
				10.905	[68]
		*K. pneumoniae*	ESBL^+^ 14-17	1.363	[54]
				10.905	[68]
			ESBL^+^ 14-18	1.363	[54]
				2.276	[68]
			ESBL^+^ 14-19	174.482	[54,68]
			*-*	11.069	[80]
			14-1	43.621	[54,68]
			14-2	21.810	[54]
			14-3	87.241	[54]
				43.621	[68]
			14-4	43.621	[54]
				21.810	[68]
			ATCC 700603 ESBLs^+^	1.364	[55]
				1.380	[51]
			ESBLs^-^	≤0.082	[55]
			ESBLs^-^	0.170	[51]
			12-4	0.082	[69]
			12-7	1.363	[69]
		*P. aeruginosa*	ATCC 27853	2.726	[54,55,68]
				5.540	[51]
			14-9	1.363	[54]
				2.726	[68]
			14-11	5.453	[68]
			14-14	5.453	[54]
			14-15	5.453	[54,68]
			14-16	5.453	[54]
			14-19	5.453	[68]
			12-12	1.363	[69]
			12-14	87.241	[69]
			12-20	21.810	[69]
		*M. morganii*	ATCC 25830	≤0.083	[55]
				≤0.080	[51]
		*P. mirabilis*	13-1	0.166	[55]
			ATCC 49565	≤0.080	[51]
		*P. rettgeri*	ATCC 31052	≤0.080	[51]
				≤0.83	[55]
		*P. vulgaris*	ATCC 29905	≤0.080	[51]
				≤0.083	[55]
		*S. maltophilia*	ATCC 13636	2.767	[55]
				1.380	[51]
		*S. marcescens*	ATCC 21074	0.350	[51]
				0.356	[55]
		*S. pneumoniae*	ATCC 49619	0.345	[26]
			12-18	2.535	[26]
	Sparifloxacin	*E. coli*	F-50	0.484	[44]
		*P. aeruginosa*	ATCC 9027	0.484	[44]
	Gatifloxacin	*E. coli*	ATCC 700603	0.160	[71]
			NCDC 134	266.387	[42]
		*K. pneumoniae*	ATCC 25922	2.664	[71]
		*P. aeruginosa*	NCDC 105	106.555	[42]
	Moxifloxacin HCl	*A. baumannii*	ATCC 19606	0.972	[50]
		*E. coli*	ATCC 25922	0.137	[68]
				<0.018	[69]
				0.037	[24]
				<1.370	[28]
			NR 17663	0.037	[24]
			NR 17666	0.075	[24]
			NR 17661	79.715	[24]
			12-6	1.142	[69]
			12-11	36.539	[69]
			ESBL^+^ 12-14	36.539	[69]
			ESBL^+^ 14-1	4.567	[68]
			ESBL^+^ 14-2	36.539	[68]
			14-1	18.269	[68]
			14-2	36.539	[68]
		*K. pneumoniae*	ATCC 13883	<1.370	[28]
			ESBL^+^ 14-17	18.269	[68]
			ESBL^+^ 14-18	2.284	[68]
			ESBL^+^ 14-19	146.155	[68]
			14-1	18.269	[68]
			14-2	18.269	[68]
			14-3	73.077	[68]
			14-4	18.269	[68]
			12-4	0.069	[69]
			ESBL^+^ 12-7	1.142	[69]
		*S. pneumoniae*	ATCC 49619	0.137	[26]
			12-18	1.142	[26]
		*P. aeruginosa*	ATCC 27853	4.567	[68]
			ATCC 43288	11.601	[28]
			14-9	9.135	[68]
			14-11	36.539	[68]
			14-15	36.539	[68]
			14-16	18.269	[68]
			14-19	2.284	[68]
			PA01	7.722	[50]
			12-12	4.567	[69]
			12-14	36.539	[69]
			12-20	18.269	[69]
		*Y. pseudotuberculosis*	ATCC 911	<1.495	[28]

ZOI: Zone of Inhibition; NZ: No Zone; ND: Not Detected; *Acinetobacter baumannii* (*A. baumannii*); *Acinetobacter calcoaceticus* (*A. calcoacetius*); *Acinetobacter haemolyticus* (*A. haemolyticus*); American Type Culture Collection (ATCC); *Citrobacter freundii* (*C. freundii*); China Center of Industrial Culture Collection (CMCC); *Enterobacter aerogenes* (*E. aerogenes*); *Enterobacter cloacae* (*E. cloacae*); *Escherichia coli* (*E. coli*); Extended spectrum beta-lactamases (ESBL); *Helicobacter pylori* (*H. pylori*); *Klebsiella pneumonia* (*K. pneumonia*); *Moraxella catarrhalis* (*M. catarrhalis*); *Morganella morganii* (*M. morganii*); Nigeria Centre for Disease Control (NCDC); *Providencia rettgeri* (*P. rettgeri*); *Pseudomonas aeruginosa* (*P. aeruginosa*); *Proteus mirabilis* (*P. mirabilis*); *Proteus vulgaris* (*P. vulgaris*); *Serratia marcescens* (*S. marcescens*); *Stenotrophomonas maltophilia* (*S. maltophilia*); *Streptococcus pneumoniae* (*S. pneumoniae*); *Yersinia pseudotuberculosis* (*Y. pseudotuberculosis*).

**Table 5:** Fluoroquinolones’ antimycobacterial activity.

Fluoroquinolone		Mycobacterium Bacteria	Strain	MIC (mM)	Reference
Generation	Name				
Second Generation	Norfloxacin	*M. smegmatis*	ATCC 607	16.503	[28]
				No activity	[46]
	Ciprofloxacin	*M. tuberculosis*	36.216–51.307		[63]
			MTB H_37_Rv	MIC_90_ 1.780	[27]
				3.018	[81]
			MTB H37Rv ATCC 27294	0.755	[26,68]
			MDR-TB	6.036	[81]
			MDR-MTB 6133 resistant to INH and RFP	0.377	[26]
			MDR-MTB 11277 resistant to INH and RFP	0.377	[26]
			*M. vaccae* IMET10670	0.470	[58]
		*M. smegmatis*	ATCC607	>120.721	[28]
Cipro HCl		*M. smegmatis*	ATCC607	>109.052	[28]
Third Generation	Levofloxacin	*M. tuberculosis*	H_37_RV 76?	1.384	[65]
			MTB H37Rv ATCC 27294	0.692	[26,68]
			MDR-MTB 6133 resistant to INH and RFP	0.377	[26]
			MDR-MTB 11277 resistant to INH and RFP	0.692	[26]
			R2012-123 (pan-sensitive)	0.692	[65]
			MDR-TB	ND	[75]
		*M. abscessus*	5.535		[24]
		*M. chelonae*	5.535		[24]
		*M. fortuitum*	0.346		[24]
		*M. avium*	ND		[75]
		*M. terrae*	ND		[75]
		R-2012-59 (MDR)	0.692		[65]
		R-2012-97 (XDR)	22.138		[65]
		*M. abscessus*	ATCC19977	>88.552	[65]
		*M. chelonae*	ATCC35752	1.384	[65]
		*M. fortuitum*	ATCC06841	0.346	[65]
	Moxifloxacin	*M. tuberculosis*	H37Rv ATCC27294	0.311	[65]
			MTB H_37_Rv	0.228	[82]
			MDR-TB	0.274	[82]
			R2012-123 (pan-sensitive)	0.137	[65]
		*M. smegmatis (MXF HCl)*	ATCC607	>91.347	[28]
		Antituberculosis		0.440	[28]
		R-2012-59 (MDR)		≤0.069	[65]
		R-2012-97 (XDR)		4.567	[65]
		*M. abscessus*	ATCC19977	>73.077	[65]
		*M. chelonae*	ATCC35752	0.571	[65]
		*M. fortuitum*	ATCC06841	0.137	[65]

ND: Not determined; Mycobacterium abscessus (Mycobacterium abscessus); Mycobacterium avium (M. avium); Mycobacterium chelonae (M. chelonae); Multi drug resistant Tuberculosis (MDR-TB); Mycobacterium fortuitum (M. fortuitum); Mycobacterium smegmatis (M. smegmatis); Mycobacterium terrae (M. terrae); Mycobacterium tuberculosis (MTB).

**Table 6:** Fluoroquinolones’ antifungal and anticancer activity.

Fluoroquinolone		Fungi and Cancer	Strain	Inhibitory Effect	Reference
Generation	Name				
Second Generation	Norfloxacin	*C. albicans*	ATCC 60193	No zone of inhibition	[28]
		*S. cerevisiae*	RSKK 251	No zone of inhibition	
	Ciprofloxacin	*A. clavatus*	No zone of inhibition		[86]
		*C. albicans*	ATCC 90873 amphotericin B-resistant	MIC 97.784 μM	[34]
		*C. albicans*	ATCC 60193	No zone of inhibition	[86]
		*T. brucei*	427/421	MIC 100 μM GI_50_ 30.9 ± 3.3 μM	[66]
		Lung adenocarcinoma	A549	MIC 50 μM	[61]
		Colon cancer	HCT-116	MIC 50 μM	[61]
		Breast cancer	MCF-7	MIC 50 μM	[61]
		HEPG2, liver hepatocellular carcinoma cells	ATCC HB-8065	IC_50_ ≥ 1207.211 μM	[50]
		Vero, kidney epithelial cells	ATCC CCL-81.	IC_50_ ≥ 1207.211 μM	[50]
		Human primary colon cancer	(SW480)	IC_50_ 160.4 ± 6.7 μM	[48]
		Human metastatic colon cancer	(SW620)	IC_50_ 200.4 ± 4.9 μM	[48]
		Human metastatic prostate cancer	(PC3)	IC_50_ 101.4 ± 3.6 μM	[48]
		Human immortal keratinocyte cell line from adult human skin	(HaCaT)	IC_50_ 222.1 ± 5.2 μM	[48]
		LDH release	HaCaT	LDH release % 4.6% at 60 μM 4.2% 40 μM 3.9% 20 μM 3.2% 10 μM	[48]
		LDH release	SW480	LDH release % 15% at 60 μM 14.5% at 40 μM 14.2% at 20 μM 12% at 10 μM	[48]
		LDH release	SW620	LDH release % 9.3% at 60 μM 9.1% at 40 μM 8.9% at 20 μM 8.1% at 10 μM	[48]
		LDH release	PC3	LDH release % 18% at 60 μM 17.5% at 40 μM 16.5% at 20 μM 14% at 10 μM	[48]
		Urease inhibitory activity	94.32 μM		[78]
		HL-60	MIC > 100 μM GI_50_ > 100 μM		[66]
		Selectivity	MIC > 1 μM ratio GI_50_ > 3.2 μM ratio		[66]
		L929	GI_50_ >100 ± n.d. μM		[66]
		HeLa	GI_50_ 560 ± 22.6 μM		[66]
		DNA gyrase	IC_50_ 0.15 μM		[66]
		Topoisomerase IV	4.00 μM		[66]
		Cytotoxicity	>100 μM		[27]
	Cipro HCl	*C. albicans*	ATCC 60193	No inhibition	[28]
		*S. cerevisiae*	RSKK 251	No inhibition	[28]
Third Generation	Levofloxacin	Vero Cells	CC_50_ > 276.73 μM		[70]
		A549	76.3 ± 6.51 μM		[87]
		HepG2	>100 μM		
		MCF-7	64.2 ± 5.67 μM		
		PC-3	>100 μM		
		HeLa	71.1 ± 4.98 μM		
		MCF-10A (Human breast epithelial cell line)	>100 μM		
	Moxifloxacin	*S. cerevisiae*	RSKK 251	No inhibition	[28]
		HEPG2, liver hepatocellular carcinoma cells	ATCC HB-8065	≥ 996.435 μM	[50]
		Vero, kidney epithelial cells	ATCC CCL-81	≥ 996.435 μM	[50]

*Micrococcus luteus* (*M. luteus*); *Candida albicans* (*C. albicans*); *Saccharomyces cerevisiae* (*S. cerevisiae*); *Aspergillus clavatus* (*A. clavatus*); *Trypanosoma brucei* (*T. brucei*); lactate dehydrogenase (LDH); The half maximal inhibitory concentration (IC_50_). Minimum inhibitory concentration (MIC); Concentration causing 50% cell growth inhibition (GI_50_).
